# Germ Warfare? Strategies for Reducing the Spread of Antibiotic Resistance

**DOI:** 10.1289/ehp.121-a255

**Published:** 2013-08-01

**Authors:** Carol Potera

**Affiliations:** Carol Potera, based in Montana, has written for *EHP* since 1996. She also writes for *Microbe*, *Genetic Engineering News*, and the *American Journal of Nursing*.

A growing body of literature describes how human pathogens in the environment acquire antibiotic resistance genes (ARGs), a process potentially boosted by selection pressure from antibiotics. Contaminated water and soil may then maintain and spread these antibiotic-resistant bacteria (ARB) and ARGs. A review in this issue of *EHP* examines strategies for reducing environmental pollution with antibiotics, ARBs, and ARGs from various sources, including traditional agriculture, aquaculture, wastewater treatment plants (WWTPs), pharmaceutical manufacturers, and hospitals.^1^

“This information needs to reach a wider audience, especially in the medical and public health fields,” says coauthor Amy Pruden, a professor of civil and environmental engineering at Virginia Tech in Blacksburg.

Limiting antibiotics in animal production is the most direct way to control environmental ARB and ARGs, according to the authors.^1^ After Denmark banned antibiotics as animal growth promoters in 1998, investigators found marked reductions of antibiotic resistance. During the period 1997–2000, erythromycin resistance in *Enterococcus faecium* fell from 76% to 13% in Danish broiler chickens and from 90% to 47% in pigs, suggesting that regulations can be useful in reversing antibiotic resistance in food animals.^2^ In the United States, 70% of antibiotics are given to farm animals, largely to promote growth, not treat disease.

Good animal husbandry practices, such as low animal density and good nutrition, keep animals healthy and reduce the need for antibiotics.^3^ Immunizing farm animals and fish with inexpensive vaccines aimed at major animal pathogens would further limit the need for antibiotics.^1^ Adoption of salmon vaccines in Norway facilitated a 99% reduction in antimicrobial use between 1987 and 2007, and fish production soared from 350,000 to 850,000 metric tons during the same time.^4^ Overall, “there is a need for cheap and effective animal vaccines,” says coauthor Joakim Larsson, a professor of environmental pharmacology at the University of Gothenburg, Sweden.

Antibiotics, ARB, and ARGs from farm animals, households, hospitals, and drug manufacturers often end up at WWTPs. These facilities are designed primarily to remove solid organic matter, nitrogen, and phosphorus, although some also apply disinfectants to kill bacteria.^1^^,^^5^ WWTP processes secondarily remove some ARB and ARGs, although with some treatment methods ARGs have been shown to rebound during subsequent treatment.^6^ At least 56 antibiotics from 6 drug classes have been detected in treated sewage.^7^

WWTPs hold promise as a critical point for removing ARB and ARGs by biodegradation, adsorption, chemicals, and other effective and economical modifications. “More research is needed to identify which treatments are most effective to better design WWTP operations,” says Pruden.

**Figure:**
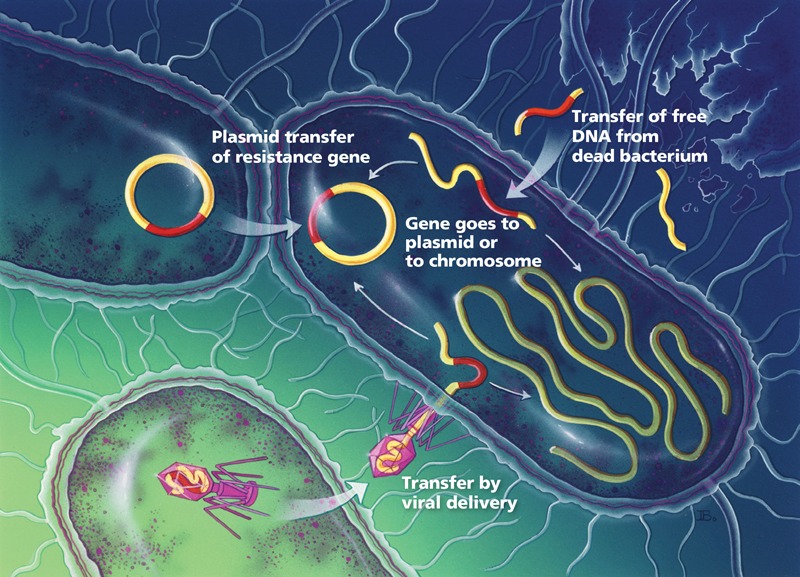
Bacteria acquire resistance by exchanging conjugative plasmids (circular units of DNA), by acquiring DNA released from dead cells, and by transferring resistance genes packaged in viruses. © Bryson Biomedical Illustrations, Inc.

Certain drug manufacturing hubs have been identified as “hot spots” that release high levels of antibiotics, ARB, and ARGs into surface, ground, and drinking water.^8^ The authors write that “a range of treatment technologies” will be necessary to address industrial production. However, they add, it’s not all about identifying or developing technical solutions—it also is important to create incentives to apply these technical solutions. As an example, they cite Sweden’s implementation of new environmental criteria in the procurement and reimbursement systems hospitals use to purchase medicines.^1^

“All solutions must start with changes in local practices and then be implemented at a global scale, or we will get nowhere,” says coauthor David Graham, a professor of ecosystems engineering at Newcastle University, United Kingdom. The problem of antibiotic resistance “will only be reduced if we change our behavior at all fronts,” Graham says. “Greater control of the medical and agricultural uses of antibiotics must be coupled with greater control of our wastes.”

Marilyn C. Roberts, a professor of environmental and occupational health sciences at the University of Washington in Seattle, commends the review authors for presenting “the big picture,” but she says providing specific details about what individuals can do would have been a valuable addition. For example, people could demand that food labels state whether food was grown with antibiotics and list the levels of antibiotic residues in a food. “This information would allow consumers to choose not to select products that use antibiotics and/or have measurable amounts of antibiotic residues,” Roberts says.

Antibiotic resistance is now making an appearance on Capitol Hill. A bill titled Strategies to Address Antimicrobial Resistance (STAAR) was reintroduced to the U.S. House of Representatives in June 2013.^9^ STAAR was originally introduced in both chambers in 2007 but died in committee.^10^^,^^11^ The House bill was reintroduced in 2009 but once again failed. The legislation would establish an Office of Antimicrobial Resistance and strengthen funding for related research.
